# Immuno-SPR biosensor for the detection of *Brucella abortus*

**DOI:** 10.1038/s41598-023-50344-5

**Published:** 2023-12-21

**Authors:** Laura Pasquardini, Nunzio Cennamo, Francesco Arcadio, Chiara Perri, Alessandro Chiodi, Girolamo D’agostino, Luigi Zeni

**Affiliations:** 1 Indivenire Srl, Via Sommarive 18, 38123 Trento, Italy; 2 Department of Engineering, University of Campania “Luigi Vanvitelli”, Via Roma 29, 81031 Aversa, Italy; 3 Moresense Srl, Filarete Foundation, Viale Ortles 22/4, 20139 Milan, Italy

**Keywords:** Biotechnology, Health care, Engineering, Optics and photonics

## Abstract

A proof of principle biosensor for the *Brucella abortus* recognition onsite is presented. The system is based on a plasmonic optical fiber probe functionalized with an oriented antibody layer immobilized on a short polyethyleneglycol (PEG) interface through carbodiimide chemistry and protein G as an intermediate layer. The biosensor is inserted in a holder built in 3D printing technology, obtaining a custom holder useful for housing the sample to be measured and the equipment. The removable sensor chip is a low-cost Surface Plasmon Resonance (SPR) platform based on D-shaped plastic optical fibers (POFs), built-in in 3D printed connectors, used here for the first time to detect bacteria via a bio-receptor layer specific for its membrane protein. The performances of the biosensor in *Brucella abortus* recognition are tested by using two different SPR-POF probes combined with the same bio-receptor layer. The best sensor configuration has presented a sensitivity at low concentrations of one order of magnitude greater than the other. A limit of detection (LoD) of 2.8 bacteria/mL is achieved well competitive with other systems but without the need for amplification or special sample treatments. Specificity has been tested using Salmonella bacteria, and reproducibility, regenerability and stability are moreover evaluated. These experimental results pave the way for building an efficient and specific biosensor system for *Brucella abortus* detection onsite and in a few minutes. Moreover, the proposed POF-based SPR biosensor device, with respect to the already available technologies, could be a Point-of-care-test (POCT), simple to use, small-size and portable, low-cost, don’t necessary of a microfluidic system, and can be connected to the Internet (IoT).

## Introduction

Brucellosis is a zoonotic disease caused by bacteria of the genus *Brucella*. It is mainly associated with animals, like cows and sheep, but can also infect humans^1,2^. People can become infected by eating or drinking unpasteurized/raw dairy products or through direct contact, mainly with the tissues of infected animals (like placenta or aborted tissues).

The main effects of this disease in animals can be direct and indirect: inside direct effects, it can be listed the losses due to animal abortion, the premature death of infected animals, reduced milk production, reduced fertility, the vertical transmission to offspring, and the high veterinary treatment costs. The indirect effects are the costs related to the implementation of a veterinary infrastructure and the vaccination campaigns and the high negative economic impact deriving from the direct effects^3^. In humans, brucellosis typically manifests malaise, fatigue, arthritis, and fever that can result in chronic symptoms.

Even if great progress in disease control has been made in many areas of the world, there are still regions where brucellosis is endemic. Recently Islam et al. reviewed almost 70 studies from 2001 to 2022, analyzing the incidence of Brucella species in milk and milk products and the prevalence of human brucellosis resulting from consuming contaminated milk^4^. The incidence of the disease is recorded in poorly developed regions like Latin America, North and East Africa, the Middle East, South and Central Asia, but also in the southern countries close to the Mediterranean basin, where the disease still persists. For instance, in the south part of Italy, mainly in Campania, Calabria, Puglia and Sicily, Brucellosis is persistent, causing a big issue for the Italian cattle production system^5^.

The commonly used techniques for the disease's diagnosis are Rose Bengal plate, complement fixation and agglutination, but these techniques can have low sensitivity, poor specificity, and are time‐consuming. The recommended initial test for *Brucella* infection identification from the Center for Disease Control and Prevention^6^ is based on the agglutination test used to verify the presence of anti-brucella antibodies in serum: a value ≥ 120 I.U./mL is considered positive for the infection. Other diagnostic assays are based on polymerase chain reaction (PCR), which requires microorganism isolation, identification, and nucleic acid amplification-based with the need for specialized people to perform the test and interpret the results^7^. Therefore, there is a need to develop sensitive and specific biosensors to identify the bacteria to contain the infection rapidly. The detection of bacteria using immunosensors is quite spread^8–11^, and some examples are related to the detection of *Brucella* sp.^2,7,12–16^. Apart of some examples based on electrochemical detection^2,14,15,17^, most reported systems are based on optical detection. Some of them use metal nanoparticles^13,16,18^, or the microscope visualization^12^, and more complex systems are instead based on a lateral flow assay combined with loop-mediated isothermal amplification^19^ or on the hybridization on ionic layer-by-layer films coupled to a long-period grating optical fiber^20^. Other sensor systems are based on pathogen enrichment, DNA extraction and hybridization detection through ring resonators^21^ or phage-functionalized nanostructures for SERS detection^1^ or quartz crystal microbalance^22^.

Modified plastic optical fibers (POFs) have been used to realize several simple and highly sensitive biosensors for different application fields^23^, for instance, by using plasmonic phenomena combined with several types of bio-receptor layers (e.g., antibodies and aptamers)^24,25^. This sensing approach is used to detect numerous substances using POF-based SPR biosensors, and recently, the SARS-CoV-2 virus has also been detected via its spike protein by specific aptamers and molecularly imprinted polymers (MIPs)^26^.

The high spreading of the infection both of bacteria aerosol and viruses encourages the development of strategies for the on-site detection of airborne pathogens performing an early-stage infection detection and reducing the risk of the spreading on large scale. In a recent review, Qiu and co-workers^27^ underliened the relevance of biosensors approaches and point-of-care devices in the rapid and accurate detection of pathogens that can be crucial in the disease transmission control as also assessed by Talebian et al.^28^ An example of a portable point of care for the detection of bacteria is reported by Tokel et al^29^.

In this work, the bacteria detection via its membrane protein is presented for the first time by exploiting SPR D-shaped POF biosensors. More specifically, this work presents a proof of principle of a novel Brucella sensor system, exploiting a disposable POF biosensor based on a specific antibody for *Brucella abortus* and the SPR method. In order to improve the biosensor performances for the recognition of the Brucella at low concentrations efficiently, the combination of a POF-based doped SPR sensor^30^ with an oriented antibody immobilization^31^ is presented. The sensor's sensitivity, specificity, reusability, reproducibility and stability are evaluated, confirming the excellent performance of the proposed optical biosensor system.

## Results and discussion

**Figure 1:**
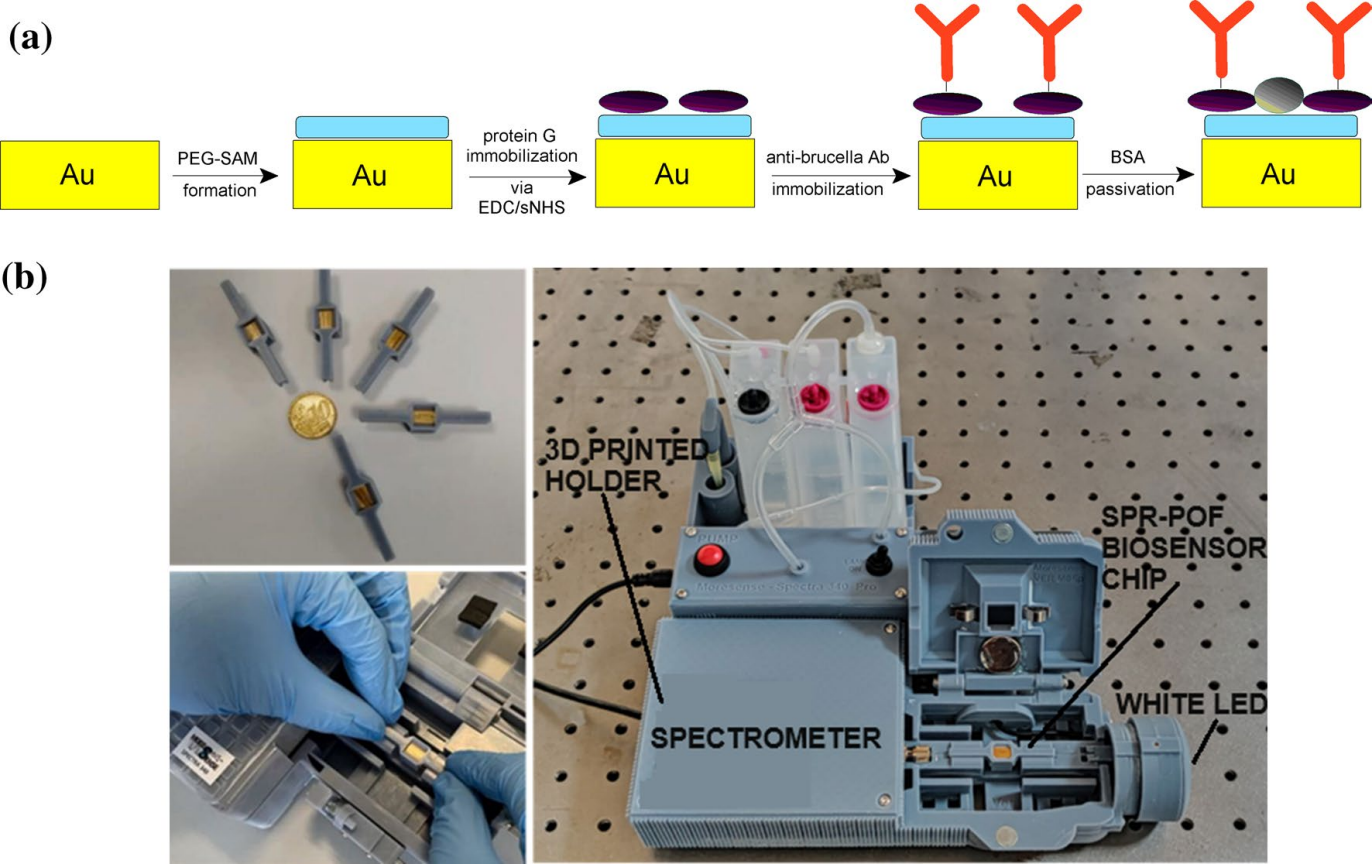
(**a**) Scheme of the functionalization process, (**b**) pictures of the POF-based SPR biosensor and the experimental setup (3D-printed holder with the equipment used for the experiments).

Gold functionalization has been optimized and widely characterized by physical–chemical techniques, as reported in our previous work^31^. The immobilization protocol (Fig. 1a) and the optimal conditions (oriented antibody layer at the proper density) have been selected^31^ and here used for functionalizing and testing the SPR POF-based platforms monitored by exploiting the 3D-printed custom holder containing a spectrometer and a white light source inside (Fig. 1b).

The POF-based SPR probe reported in Fig. 1b was developed during the time by the collaboration between the University of Campania Luigi Vanvitelli and its SPIN-OFF Moresense srl. In particular, by starting from the SPR-POF probe developed by Cennamo et al.^32^, a novel SPR-POF chip has been developed by using a PMMA doping approach^30^ instead of a photoresist buffer layer (under the gold nanofilm) to improve the plasmonic phenomena, and 3D-printed connectors, integrated with a measuring cell, are used instead of removable SMA connectors (see Fig. 1b). Exploiting the cell built-in in the chip connectors, the *Brucella abortus* detection can be obtained by dropping and doesn’t require a microfluidic system. The experimental setup reported in Fig. 1b is around 25 cm × 25 cm and is about 1 kg in weight.

### Brucella sensor performances

**Figure 2:**
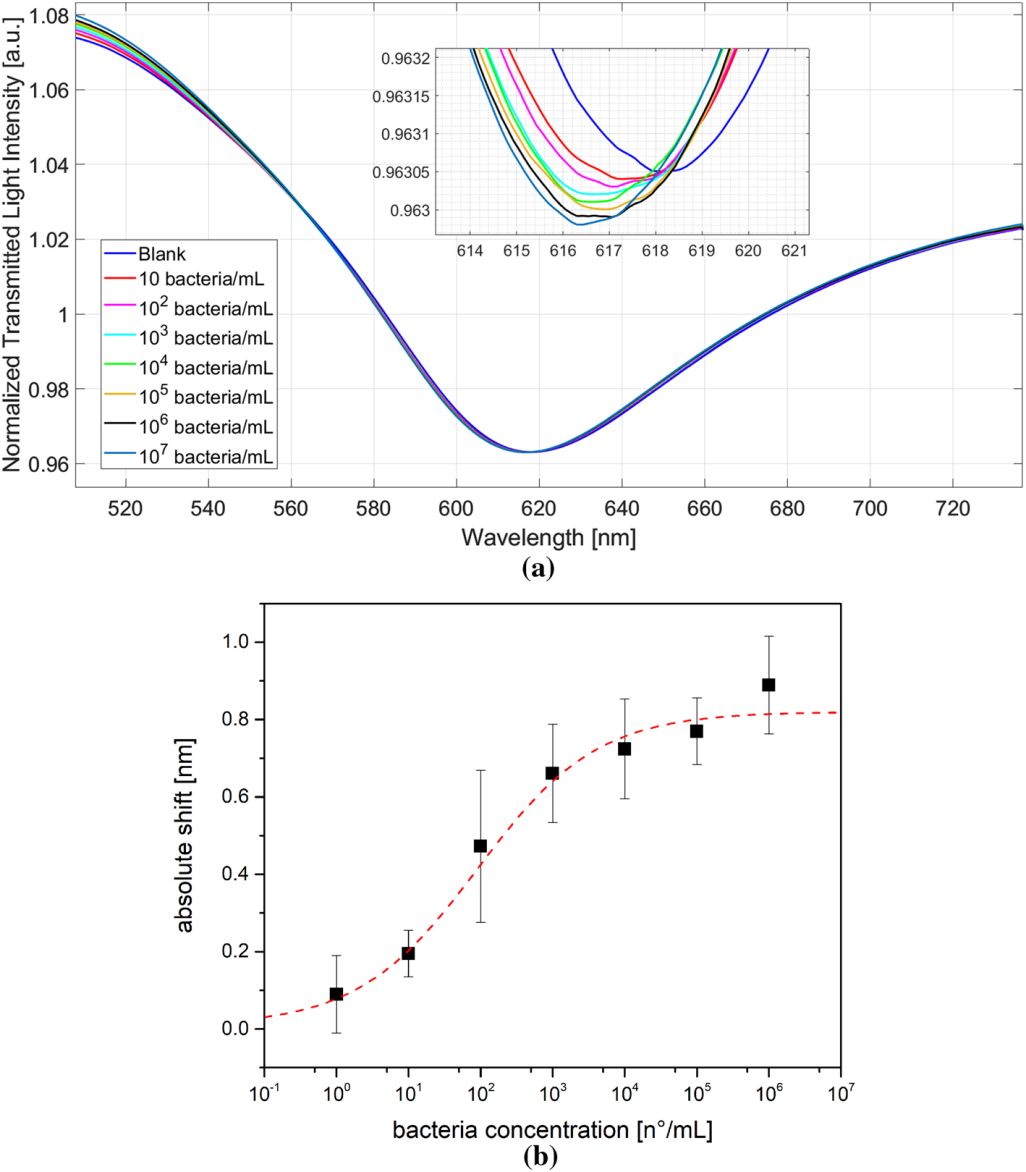
(**a**) Transmission spectra normalized on air after *Brucella* recognition at different dilutions; (**b**) absolute shift as function of the bacteria concentration. Data are reported as mean value of 7 sensors and error bars are reported as standard deviation. Hill function is used for data fitting (correlation coefficient of 0.98).

The *Brucella* bacteria are incubated at different concentrations on the immunosensor surface (using the 3D-printed measure cell reported in Fig. 1b), and the resonance shift is acquired after the incubation and washing steps (Fig. 2a). More specifically, all the spectra reported in Fig. 2a, obtained at different bacteria concentrations between 1 and 10^6^ bacteria/mL, are acquired with buffer as a bulk solution after 5 min of incubation with the sample and twenty washing steps by buffer. So, the SPR spectra are acquired with the same bulk solution and after an extensives washing steps to remove the non-specific binding with the surface. In other words, the dose–response curve is, therefore, the result of the refractive index variation induced by bacteria binding on the functionalized sensor.

By considering the kind of bio-receptor used in this work, as shown in the SPR spectra reported in Fig. 2a, when the analyte-receptor binding occurs, the refractive index of the receptor layer decreases, and the resonance wavelength decreases (blueshift), similarly to other receptors combined with the same SPR-POF probe^24,33–35^.

In order to obtain the sensor's calibration curve (dose–response curve), the average values of obtained resonance wavelengths (results achieved in several similar measurements, carried out by ten similar biosensors) with the relative standard deviations (error bars) are reported in Fig. 2b. The fitting of the data shown in Fig. 2b is obtained using the Hill formula, recalled in the following equation:

1 $$\Delta \lambda = \Delta \lambda_{0} + \left( {\Delta \lambda_{{{\text{MAX}}}} - \Delta \lambda_{0} } \right) * c^{n} / \left( {k^{n} + c^{n} } \right)$$

where ${\Delta \lambda }_{0}$ is the initial parameter (obtained by the blank solution), ${\Delta \lambda }_{{\text{MAX}}}$ is the final parameter (relative to the saturation value), *n* indicates the Hill coefficient, *k* indicates the Michaelis constant, and *c* the concentration of the analyte.

The Hill parameters obtained from the fit of the experimental values (Fig. 2b) are reported in the supplementary file (Table S1).

The Hill equation (often used to model the enzyme/substrate behavior and biosensors in general) is used to fit the data^36^: when the Hill coefficient n is 1, it turns into the classical Michaelis–Menten equation, when one analyte binds to one receptor. When n > 1, an S-shape curve and a positive cooperation are observed, meaning that more molecules bind to other distal sites on the same receptor. When n < 1, the binding of other “molecules” to other distal sites on the same receptor is unfavorable, the curve gradually approaches to a maximum value and it translates into a negative cooperation that increases the dynamic range^37^. Fitting the data in Fig. 2B with the Eq. (1), a value of n = 0.5 is obtained, suggesting a negative cooperation, i.e. when a bacterium binds through the LPS protein to one antibody, the binding of another bacterium protein is unfavored.

However, negative cooperation translates into a more sensitive response, as assessed by Ha and Ferrel^38^: developing a theoretical model and testing with DNA annealing, authors demonstrated that a negative cooperativity is characterized by a marked threshold and a high degree of ultrasensitivity on the formation of a ternary complex between a receptor and two high-affinity ligands.

Blue shifts using a similar antibody concentration are also observed by Richter, using a long period grating fiber system for the detection of T7 bacteriophages^39^.

To calculate the limit of detection (LoD) of the tested sensors, a range between 1 and 10^4^ bacteria/mL is used in order to avoid the not specific binding that occurs at high concentrations due to the bacteria precipitation. In particular, the Eq. (2) is used to obtain the LoD, as here reported:

2 $${\text{LoD}}=\frac{3*\left({\text{St}}.{\text{Dev}}.\right) {\Delta \lambda }_{0}}{\frac{\left({\Delta \lambda }_{{\text{MAX}}}-{\Delta \lambda }_{0}\right)}{k}}$$

where ${\Delta \lambda }_{0}$, ${\Delta \lambda }_{{\text{MAX}}}$ and *k* are the parameters obtained from the fit using Eq. (1). Using the values obtained and reported in Table S2 (standard platform), a LoD of 2.8 bacteria/mL in buffer is achieved. This value is excellent when compared with other Brucella biosensors, as reported in Table 1, especially considering that most of the reported LoDs are expressed as CFU/mL (colon forming unit) and one single CFU can be formed by several bacteria, and that the presented system is based on a label-free direct measure without the need of amplification.

**Table 1:** Comparison of the developed biosensor for *Brucella* detection.

1	Method	Technique	LoD	Reference
2	Phage-functionalized nanostructures	SERS	≈ 10^4^ bacteria	Rippa et al.^1^
3	Ionic LBL (layer-by-layer) film coupled to a long-period grating optical fiber modified with a biotin-modified nucleotide probe complementary to a specific Brucella region	Attenuation of light transmission	> 10^2^ bacteria	McCutcheon et al.^20^
4	Lateral flow assay combined with loop mediated isothermal amplification (LAMP)	Optical	100 fg (bacterial DNA)	Li et al.^19^
5	Blue-SiNPs and paramagnetic nanoparticles (PMNPs)	Optical	4.5 × 10^2^ CFU/mL	Shams et al.^16^
6	ZnO-NPs coniugated with antibodies	Electrochemical	- Tested 1000 bacteria/mL	Wahab et al.^2^
7	Immuno-gold nanoparticle-modified screen-printed carbon electrodes	Electrochemical	4 × 10^4^ CFU/mL	Wu et al.^15^
8	Aptamer modified QCM chip	QCM	1 × 10^3^ CFU/mL	Bayramoglu et al.^22^
9	Protein A-oriented antibody on silicon substrate	Optical microscopy	1 × 10^6^ CFU/mL	Baltierra-Uribe et al.^12^
10	Double antibody-modified magnetic microparticles	Absorbance	160 CFU/mL	Taheri et al.^13^
11	Pathogen enrichment, DNA extraction and hybridization detection through ring resonator	Optical	1 CFU/mL (in urine)	Zhao et al.^40^
12	Electrochemical magnetic microbeads-based biosensor (EMBIA) with dual step antibody	Electrochemical	-	Cortina et al.^17^
13	Hybridization assays with metal nanoparticles and aggregation in salt-conditions	Colorimetric	1 × 10^3^ CFU/mL	Pal et al.^18^
14	Antibody-modified electrode	Electrochemical	5.12 × 10^2^ CFU/mL	Chen et al.^14^
15	Antibody-modified optical fiber (standard probe)	SPR	2.8 bacteria/mL	Present study

In order to evaluate the platform performance and increase the binding sensitivity, a doped POF platform^30^ is used. A Fe_2_O_3_ doping procedure of the POF's core (of the PMMA) is adopted to enhance the plasmonic phenomena and the bulk sensitivity through an enhancement of the evanescent field and subsequently the SPR phenomenon that is achieved chemically modifying the first nanometers of the PMMA surface, as reported in our recent work^30^. This doped PMMA nanolayer presents a refractive index major than the PMMA in order to move up the electromagnetic fields and improve the SPR phenomena, in a similar way to the photoresist buffer layer (Microposit S1813) used on the exposed PMMA in the D-shaped POF region, under the plasmonic gold nanofilm^32^. This high-sensitive SPR-POF probe based on PMMA’s doping has been tested for the first time in bacteria sensing in this work.

**Figure 3:**
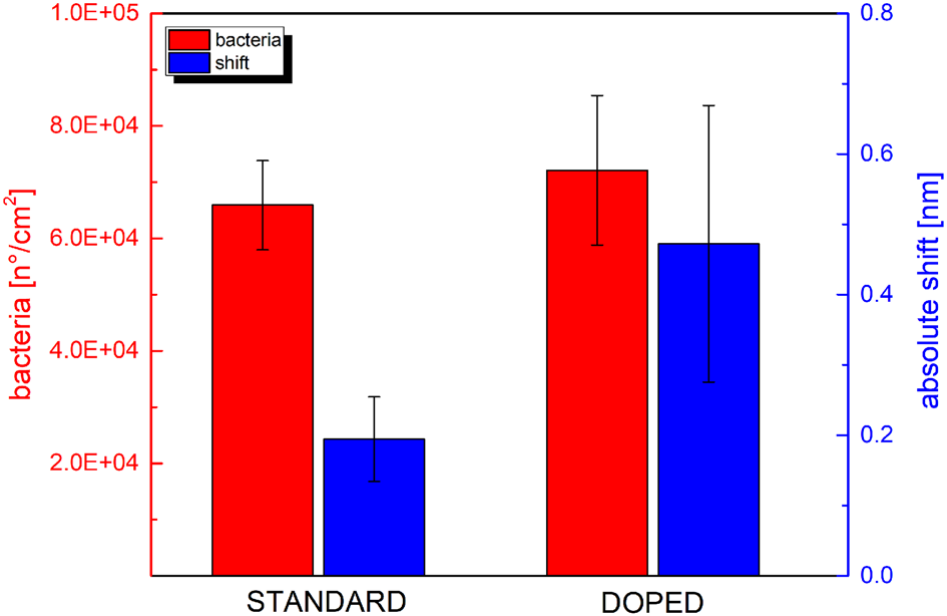
Comparison of the resonance absolute shift obtained with standard POF-based sensors versus doped-POF ones against the detection of 10^4^ bacteria/mL of *Brucella* (blue bars). The number of bacteria detected in fluorescence is also reported (red bars). Data are reported as mean values of at least two sensors in independent experiments. Error bars represent the standard deviation.

As reported in Fig. 3, besides the number of bacteria detected in fluorescence is similar (red bars), a clear difference between doped POF and not-doped POF probes in the absolute shift at low bacteria concentration (10^1^ bacteria/mL) is recorded (blue bars). Similarly to the bulk solution refractive index measurements^30^, the biosensors based on doped POF platforms are more sensitive at low bacteria concentrations with respect to the biosensors based on standard POF platforms (without the doping step).

In order to test the specificity of the doped POF sensors, *Salmonella* is used. A range between 1 and 10^6^ bacteria/mL is spanned, and the SPR spectra are achieved. The resonance variations, obtained by exploiting a doped POF biosensor with *Brucella* and *Salmonella* in the range from 1 to 10^6^ bacteria/mL, are reported in Fig. S1, highlighting that at low concentrations no detection of *Salmonella* (resonance shift) occurs, while at high bacteria concentrations a not-specific response is observed, probably due to the precipitation of bacterial cells onto the sensor surface (due to the static incubation conditions). Moreover, the selectivity of the used bio-receptor interface with respect to the impact of non-bacterial interfering agents (e.g., bovine serum albumin) has been widely characterized in our previous work^31^.

**Figure 4:**
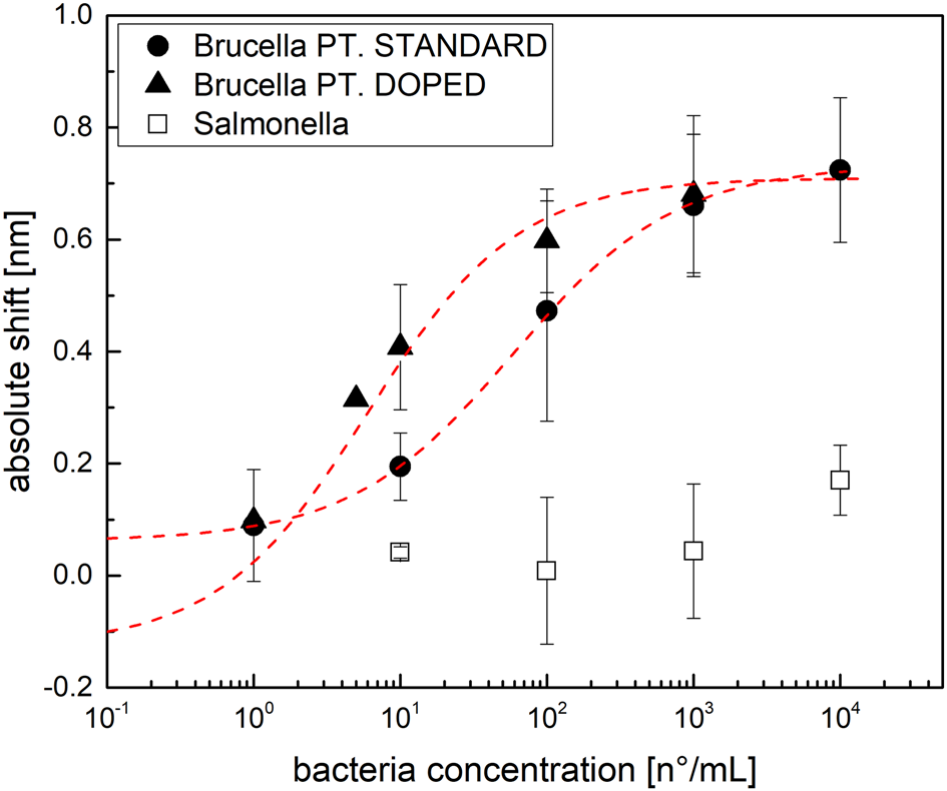
Comparison of the specificity of the sensor against *Brucella* with respect to *Salmonella* using doped POF sensors, compared to standard platforms. Data are reported as mean values of at least two sensors, and error bars represent the standard deviation. *Brucella* data are fitted using a Hill function, obtaining the parameters reported in Table S2.

Reducing the interested range at 10^4^ bacteria/mL, a clear response (resonance shift) is obtained with the specific antibody anti-Brucella, both using standard and doped POF biosensors, as reported in Fig. 4. The Hill function (Eq. 1) is used to fit this range of concentrations (parameter values are reported in Table S2), showing a good correlation both for standard (corr. = 0.99) and doped POF biosensors (corr. = 0.94) and an increase of one order of magnitude in the sensibility at low concentration (proportional to 1/k) for the doped POF biosensors is achieved (k value decreases from 59.61 to 6.03 for standard and doped platform respectively, Table S2), suggesting that the doped platforms with the enhancement in the evanescent field and in the SPR phenomena resulted more sensitive at low concentrations.

### Sensor reproducibility, reusability and stability

**Figure 5:**
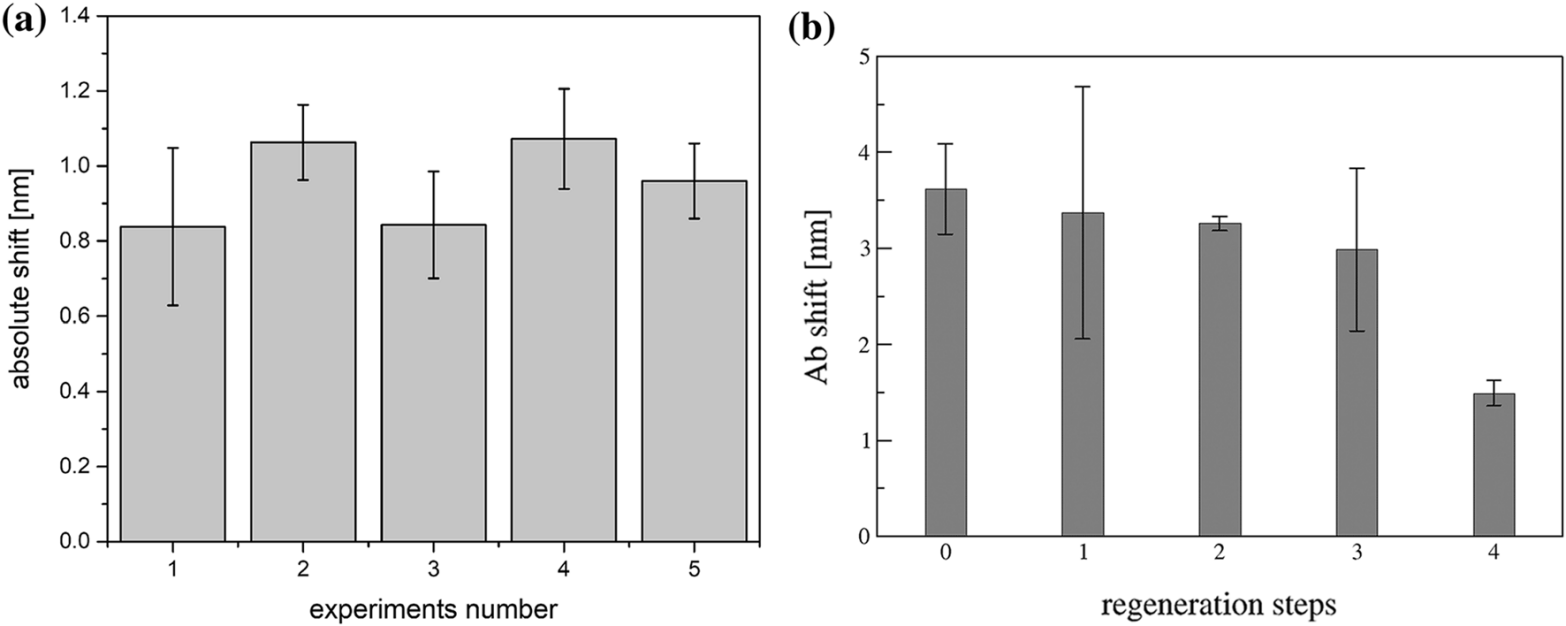
(**a**) Reproducibility of the biosensor performances in the detection of 1 × 10^4^ bacteria/mL, functionalized in five different experiments; (**b**) antibody binding on the PtG layer as a function of the regeneration of the biosensors using 10 mM glycine–HCl solution at pH 2, data are reported as mean value of two independently functionalized sensors and error bars represent the standard deviation.

The results' reproducibility is assessed by measuring different sensor chips, functionalized and tested on different days. During the experiments, at least ten biosensors have been tested in five independent experiments, obtaining an absolute resonance shift as a response at 1 × 10^4^ bacteria/mL solution equal to 0.98 ± 0.3 nm, as reported in Fig. 5a, corresponding to a bacteria concentration of (4,6 ± 2,4) × 10^4^ bacteria/cm^2^, as confirmed by fluorescence dye.

The surface can be regenerated using different solutions^41^, but the most used is based on glycine^42,43^. Here, a 10 mM of glycine–HCl pH 2 solution is applied for 5 min, destroying the binding between the antibody and the protein layer that remains covalently attached to the sensor surface. Our results suggest that up to three regeneration steps can be applied without compromising the binding of the new antibody solution (see Fig. 5b), even if the possibility of regenerate the surface is not a mandatory aspect for the proposed device, that aims to detect quickly bacteria through disposable sensors.

Moreover, to monitor the shelf life of the functional layer, the functionalized sensors are maintained at 4 °C for 25 days in dry condition or PBS buffer or in PBS + 2% w/v BSA^44^ or are aged for five days at 35 °C (corresponding to 6 months as reported by the standard protocol BS-EN 13,640 "Stability testing of in vitro Diagnostic Medical Devices")^45,46^ and then 20 days at 4 °C.

The different ageing protocols were compared by monitoring the resonance shift obtained in water solution in order to have an indication of the optimal storage condition. Figure S2 suggests that maintaining the functionalized sensors in PBS at 4 °C is the more conservative condition when compared with a freshly prepared biosensor. In comparison, the accelerated aging at 35 °C causes a resonance increase in the functional layer, suggesting a possible precipitation of antibodies on the plasmonic surface (the measured refractive index at the dielectric gold interface increased) and a possible loss of functionality.

## Conclusions

A proof of principle POF biosensor for the *Brucella abortus* detection is presented, based on an oriented antibody layer and an SPR D-shaped POF probe, monitored by a portable and low-cost instrument. The analytical parameters like LoD, specificity, reproducibility, reusability and stability are evaluated. A LoD of 2.8 bacteria/mL in buffer is achieved, and the binding sensitivity of the sensor system increases by one order of magnitude using doped POF sensors (k value decreases from 59.61 (standard) to 6.03 (doped) platform resulting in a more sensitive sensor since 1/k is related to the higher sensitivity). The measurement time is around 10 min (incubation plus washing steps). The specificity is tested using *Salmonella* as a non-specific bacteria, and the functionalization process is tested in different experiments, showing good reproducibility. The reusability of the sensor is optically evaluated, showing promising results up to three regeneration cycles and the platform's stability is optically evaluated in different conditions, showing that conservation in buffer at 4 °C is a good way to maintain the functionalized sensors. Our experimental results pave the way for building a portable, highly sensitive, and stable device for *Brucella abortus* recognition onsite. In particular, the proposed sensor system could be a POCT that does not want to substitute the currently used techniques for the official detection of Brucella, like Rose Bengal plate, complement fixation and agglutination or PCR; instead, it is aimed to provide an easy POCT to the farmer for the periodical screening of his herd, in order to isolate the ill animal quickly and safe in this way the other animals.

## Methods

### Materials and reagents

Gold substrates are prepared by depositing 10 nm of titanium on a silicon substrate (100), followed by 100 nm of gold purchased from MicroFabSolution srl (Trento, Italy). M-dPEG®8-Thiol (mPEG) and HS-dPEG™ (12)-COOH (PEG-COOH) for the self-assembled monolayer (SAM) are purchased from Stratech (United Kingdom) and from Iris Biotech GmbH (Marktredwitz, Germany) respectively. 1-ethyl-3-(3-dimethylaminopropyl) carbodiimide hydrochloride (EDC, 22980) and N-hydroxysulfosuccinimide (Sulfo-NHS, 24,510) and Pierce recombinant protein G (PtG, 21193) are purchased from Fisher Scientific Italia (Milan, Italy), while the fluorescent derivative Alexafluor488- Protein G (P11065) is purchased from Thermo Scientific (Rockford, IL, USA). The recombinant monoclonal anti-Brucella antibody produced in mouse (Brucella abortus (Bx88)), designed to recognize the lipopolysaccharide (LPS) protein on *Brucella abortus* membrane, is purchased from Santa Cruz Biotechnology, Inc (Heidelberg, Germany). Bovine serum albumin (BSA, A7030), anti-mouse polyvalent immunoglobulins (G,A,M) − FITC, antibody produced in goat (F1010) (IgG FITC), glycine and all powders for buffers are purchased from Sigma-Aldrich s.r.l. (Milan, Italy). Brucella antigenic suspension (BIO-63241) and Salmonella typhi suspension VI (BIO-63572) are purchased from Diagnostic International Distribution spa (Milan, Italy). LIVE/DEAD™ BacLight™ Bacterial Viability and Counting Kit (L34856) purchased from Thermo Scientific (Rockford, IL, USA) is used to detect bacteria on functionalized surfaces.

### Sensor functionalization

The sensor platforms are functionalized using the optimized protocol previously set up^31^. Briefly, the sensors are first cleaned using an argon plasma for 2 min at 6.8 W to remove organic contaminants. Then, a mixture at 0.2 mM total concentration of a mPEG:PEG-COOH at 0.05:0.15 mM in MilliQ water at room temperature for one hour is applied. After washing the sensors in MilliQ water, an EDC/sNHS ratio of 40/10 mM for 30 min in 50 mM of MES buffer pH 5.5 in an orbital shaker is used to activate the carboxylic groups. A Protein G layer is formed, incubating 50 ng/μL of protein in PBS (10 mM phosphate buffer, 138 mM NaCl, 2.7 mM KCl, pH 7.4) for one hour and after washing in PBS the anti-brucella antibody is incubated at 10 ng/μL for one hour in 50 mM of MES buffer pH 5.5. Finally, a passivation step in a solution of 1% w/v of BSA in PBS buffer for 30 min is performed.

### Fluorescence detection

The bacterial suspension is characterized using the protocol reported in^31^. Briefly, bacteria are stained with 5 μM of Syto®9 component of the Live/Dead kit for 15 min at dark, according to manufacturer instructions, and, using a Thoma cell counter cell, the fluorescent bacteria are quantified under the microscope, following the protocol instructions. A Leica DMLA fluorescence microscope equipped with a mercury lamp and a fluorescence filter L5 (Leica Microsystems, Germany) is used and the fluorescence is measured using a 10× magnification objective with a cooled CCD camera (DFC420C, Leica Microsystems, Germany) and analyzed with the Fiji software^47^. After the bacteria incubation on the surfaces for 5 min, a washing step in PBS is applied for ten times using the automatic pump in the setup. The quantification is obtained by converting the picture in an 8-bit image and setting a threshold and automatically counting as described in the supplementary file of our previous work^31^.

The fluorescent antibody is detected using the same experimental instrument and a 20× magnification objective.

### Sensor regeneration

The functionalized sensors are regenerated using a 10 mM glycine–HCl solution at pH 2 for 5 min in static conditions. Then, 10 washing in ultrapure water are used to remove any residual solution and a new antibody solution at 10 ng/μL for one hour in 50 mM of MES buffer pH 5.5 is applied. The effect of the regeneration step is monitored acquiring the optical transmission spectrum after the antibody incubation and after the regeneration procedure and measuring the total shift obtained after a new antibody solution incubation.

### Optical characterization

An SPR method is used to monitor the Brucella-receptor binding. The SPR probe is based on modified POFs connected to the equipment (while light source and spectrometer) by a 3D-printed custom holder, manufactured by the authors of Moresense Srl in collaboration with the other authors of University of Campania L. Vanvitelli. More specifically, the experimental setup is based on a spectrometer VIS range 500-730 nm and a white light source 400-780 nm, assembled as reported in Fig. 1b. The optical platform is based on SPR POF chip built with a custom connector exploiting additive manufacturing technology carried out by the authors of Moresense Srl in collaboration with the other authors of University of Campania L. Vanvitelli (both standard and doped POF probes). The SPR spectra are obtained by custom Software, which is user-friendly and able to connect the POCT to the internet^48^. After an incubation step with buffer for stabilizing the optical signal, the biosensor chip can be used. The bacterial suspensions are prepared, diluting the stock solution after vortex mixing, and 100 μL of the samples are dropped in the measuring cell (on the sensor surface) and incubated for 5 min. Then, 20 washing steps in the buffer are performed, and finally, a fresh PBS buffer solution is added to acquire the transmission spectrum. The SPR spectra are obtained by normalizing the acquired spectrum on the reference spectrum (spectrum obtained in air).

### Supplementary Information

Supplementary Information.

## Data Availability

All data generated or analysed during this study are included in this published article [and its supplementary information files], however raw data will be available from the corresponding author on reasonable request.
