# Supplementary material for: Immuno-SPR biosensor for the detection of Brucella abortus
Source: Sci Rep. 2023 Dec 21;13:22832. doi: 10.1038/s41598-023-50344-5 (PMC10739931; doi:10.1038/s41598-023-50344-5)
Supplement: Supplementary file 1 — Supplementary Information. [file 41598_2023_50344_MOESM1_ESM.docx]

Electronic Supplementary File

Immuno-SPR biosensor for the detection of *Brucella abortus*

Laura Pasquardini^a,b*^, Nunzio Cennamo^b^, Francesco Arcadio^b^, Chiara Perri^b,c^, Alessandro Chiodi^c^, Girolamo D’agostino^c^ and Luigi Zeni^b,*^

^a^ Indivenire srl, Via Sommarive 18, 38123 Trento, Italy

^b^ Department of Engineering, University of Campania “L. Vanvitelli”, Via Roma 29, 81031 Aversa, Italy

^c^ Moresense srl, Filarete Foundation, Viale Ortles 22/4, 20139 Milano, Italy

* Corresponding authors: [luigi.zeni@unicampania.it](mailto:luigi.zeni@unicampania.it); [l.pasquardini@indiveni.re](mailto:l.pasquardini@indiveni.re)

| Analytical parameters from fitting | Value ± standard error |
| --- | --- |
| ${\Delta\lambda}_{0}$ | 0.0**1** ± 0.11 |
| ${\Delta\lambda}_{MAX}$ | 0.82 ± 0.04 |
| n | 0.52 ± 0.17 |
| k | 90.39 ± 68.03 |
| Reduced Chi-Square | 0.219 |
| Adj. R-Square | 0.98 |

**Table S1.** Analytical parameters obtained from the fitting of data reported in Figure 2b using equation (1)

| Analytical parameters from fitting | Value ± standard error | |
| --- | --- | --- |
|  | STANDARD | DOPED |
| ${\Delta\lambda}_{0}$ | 0.06 ± 0.01 | -0.13 ± 0.41 |
| ${\Delta\lambda}_{MAX}$ | 0.73 ± 0.01 | 0.71 ± 0.06 |
| n | 0.78 ± 0.05 | 0.85 ± 0.76 |
| k | 59.51 ± 5.63 | 6.03 ±9.05 |
| Reduced Chi-Square | 0.004 | 0.005 |
| Adj. R-Square | 0.99 | 0.94 |

**Table S2.** Analytical parameters obtained from the fitting of data reported in Figure 2b from 1 to 10^4^ bacteria/ml and data for doped POF sensors of Figure 4 using equation (1).





**Figure S1.** Absolute shift on doped POF platforms at different concentration of bacteria, *Brucella* (black tringles up) or *Salmonella* (white squares).





**Figure S2.** Resonance wavelength variation of the functional layer with respect to the bare surface, with water as bulk solution, after different aging protocols: aging of the functionalized sensors for 25 days at 4°C or 5 days at 35°C and 20 days at 4°C in dry conditions or in PBS buffer or in PBS+2% w/v BSA compared with a freshly prepared sensor.
